# Chitin Synthases from *Saprolegnia* Are Involved in Tip Growth and Represent a Potential Target for Anti-Oomycete Drugs

**DOI:** 10.1371/journal.ppat.1001070

**Published:** 2010-08-26

**Authors:** Gea Guerriero, Mariano Avino, Qi Zhou, Johanna Fugelstad, Pierre-Henri Clergeot, Vincent Bulone

**Affiliations:** 1 Division of Glycoscience, School of Biotechnology, Royal Institute of Technology, AlbaNova University Centre, Stockholm, Sweden; 2 Department of Biological Sciences, University of Alberta, Edmonton, Alberta, Canada; 3 Department of Botany, Stockholm University, Stockholm, Sweden; The Sainsbury Laboratory, United Kingdom

## Abstract

Oomycetes represent some of the most devastating plant and animal pathogens. Typical examples are *Phytophthora infestans*, which causes potato and tomato late blight, and *Saprolegnia parasitica*, responsible for fish diseases. Despite the economical and environmental importance of oomycete diseases, their control is difficult, particularly in the aquaculture industry. Carbohydrate synthases are vital for hyphal growth and represent interesting targets for tackling the pathogens. The existence of 2 different chitin synthase genes (*SmChs1* and *SmChs2*) in *Saprolegnia monoica* was demonstrated using bioinformatics and molecular biology approaches. The function of SmCHS2 was unequivocally demonstrated by showing its catalytic activity *in vitro* after expression in *Pichia pastoris*. The recombinant SmCHS1 protein did not exhibit any activity *in vitro*, suggesting that it requires other partners or effectors to be active, or that it is involved in a different process than chitin biosynthesis. Both proteins contained N-terminal Microtubule Interacting and Trafficking domains, which have never been reported in any other known carbohydrate synthases. These domains are involved in protein recycling by endocytosis. Enzyme kinetics revealed that *Saprolegnia* chitin synthases are competitively inhibited by nikkomycin Z and quantitative PCR showed that their expression is higher in presence of the inhibitor. The use of nikkomycin Z combined with microscopy showed that chitin synthases are active essentially at the hyphal tips, which burst in the presence of the inhibitor, leading to cell death. *S. parasitica* was more sensitive to nikkomycin Z than *S. monoica*. In conclusion, chitin synthases with species-specific characteristics are involved in tip growth in *Saprolegnia* species and chitin is vital for the micro-organisms despite its very low abundance in the cell walls. Chitin is most likely synthesized transiently at the apex of the cells before cellulose, the major cell wall component in oomycetes. Our results provide important fundamental information on cell wall biogenesis in economically important species, and demonstrate the potential of targeting oomycete chitin synthases for disease control.

## Author Summary

Oomycete pathogens can infect many organisms relevant to the agriculture and aquaculture industries, such as potato and tomato, or fishes like salmon. *Saprolegnia parasitica* represents the most important oomycete fish pathogen that challenges the productivity of fish farms due to the lack of efficient methods for containing its development and pathogenicity. Enzymes involved in cell wall formation represent potential targets of anti-oomycete drugs. The isolation and full characterization of two genes involved in the biosynthesis of chitin, a quantitatively minor cell wall carbohydrate in *Saprolegnia*, was performed. Despite its low abundance, chitin was shown to play a key role in hyphal tip growth, which is a vital process for the micro-organism. The enzymes responsible for chitin biosynthesis were located at the apex of the hyphae and specifically inhibited by nikkomycin Z. The inhibitor provoked cell death by bursting of the hyphal tips. *S. parasitica* was more sensitive to the inhibitor than the model species *Saprolegnia monoica* used for these investigations. The data demonstrate the potential of targeting chitin synthases to control the diseases caused by *S. parasitica* and pave the way for the establishment of sustainable methods to tackle the adverse effects of the pathogen.

## Introduction

*Saprolegnia parasitica* is the most important oomycete fish pathogen [1]. This micro-organism is ubiquitous in fresh water and infects salmonids by acting as a primary pathogen [2]–[4]. The group of diseases caused by *S. parasitica* and other members of the order Saprolegniales is termed Saprolegniosis. These diseases are of great concern for the aquaculture industry and represent a serious threat to fish populations in natural habitats [1]. Multiple strains of *S. parasitica* that cause more than 90% fish mortality have been isolated [2], [5]. Due to the lack of efficient methods to control pathogenic Saprolegniales, there is a dramatic re-emergence of Saprolegniosis in aquaculture [1], [3]. This problem represents important economical loss and a serious threat to the activities of fish farmers worldwide [1], [3]. Thus, there is an important need for developing efficient and sustainable methods to stop the spread of these pathogens and their virulence. The enzymes involved in cell integrity, such as those responsible for cell wall formation, represent interesting potential targets of anti-oomycete drugs.

The architecture and physical properties of oomycete cell walls are governed by a network of cellulose microfibrils that play a similar role as chitin in the walls of fungi [6], [7]. By forming a scaffold for other abundant β-glucans, such as β-(1→3) and β-(1→6) glucans, cellulose microfibrils provide structural support to the hyphae [6]. While chitin represents the most important ultrastructural compound of fungal walls [7], a crystalline form of this polymer has been reported in a few oomycete species only, in which it never accounts for more than 0.5% of the total cell wall content [8]–[11]. Other oomycetes like *Aphanomyces euteiches* are devoid of chitin, but their cell walls contain about 10% of other GlcNAc-based carbohydrates [12]. Unlike chitin, these compounds are amorphous and soluble in water, possibly due to the occurrence of unidentified sugars different from GlcNAc in their structure [12]. As opposed to the soluble GlcNAc-based carbohydrates which contributeto cell integrity in *A. euteiches* [12], the role played by chitin in the walls of the oomycete species in which it has been detected is unknown.

Although chitin is not a typical cell wall carbohydrate of oomycetes [8]–[11], genes that encode putative chitin synthases are present in species from different genera, including species in which chitin has never been detected. For instance, analysis of the full genome of the plant pathogen *Phytophthora infestans* [13] has revealed the existence of a putative chitin synthase gene in this species, while its cell wall seems to be devoid of chitin. Similarly, partial sequences of putative chitin synthases have been isolated from *Plasmopara viticola* [14], *Phytophthora capsici* [15] and *Achlya ambisexualis* [15]. Despite their structural difference with chitin, it was proposed that the cell wall GlcNAc-based carbohydrates from *A. euteiches* are synthesized by the product of one or both putative chitin synthase genes identified in this species, but this has not been demonstrated [12]. In fact, to date there is not a single example of an oomycete “chitin synthase” gene product for which the enzymatic activity has been experimentally demonstrated. This is essentially due to the difficulty in expressing these polytopic transmembrane proteins in heterologous systems. In all cases, the function of the products of the identified genes has been assumed on the sole basis of sequence similarities with fungal or yeast chitin synthases. Chitin synthase 2 from *Saccharomyces cerevisiae* represents the only example for which the capacity to catalyze the formation of β-(1→4)-*N*-acetylglucosaminyl linkages has been demonstrated *in vitro* [16].

*Saprolegnia monoica* is the only oomycete in which both crystalline chitin and putative chitin synthase sequences have been identified [11], [15]. Isolated membranes and detergent extracts of plasma membranes from this species exhibit chitin synthase activity *in vitro* [11], [17] but there is no evidence that the detected activity corresponds to the sequences identified [15]. Preliminary work in *S. monoica* using the chitin synthase inhibitor polyoxin D suggested that chitin may be involved in hyphal growth [11]. Thus, chitin synthases may represent interesting targets for growth inhibitors of *Saprolegnia* species and possibly other oomycetes that contain chitin in their walls. The potential use of drugs that interfere with chitin biosynthesis is particularly attractive since higher plants and all vertebrates that are infected by pathogenic oomycetes are devoid of chitin synthase activity.

We describe here the isolation and functional characterization of 2 chitin synthase genes from *S. monoica*. For the first time, we have successfully expressed an oomycete chitin synthase that is catalytically active *in vitro*. The recombinant enzyme was used to confirm directly the inhibitory activity of nikkomycin Z on oomycete chitin synthases. In addition, we demonstrate that chitin biosynthesis is involved in tip growth in *Saprolegnia* and that chitin synthase activities are vital for the micro-organism. Interestingly, the economically important pathogen *S. parasitica* was shown to be more sensitive to nikkomycin Z than *S. monoica*. These findings pave the way for the establishment of sustainable methods for controlling the fish diseases caused by Saprolegniales.

## Results

### Isolation of the *SmChs* genes

Two *Chs* genes, named *SmChs1* and *SmChs2*, were identified in *S. monoica* in 1997 [15]. While the complete sequence of *SmChs2* was obtained (GenBank accession number U19946), only a short segment of 600 bp corresponding to *SmChs1* could be amplified by PCR and sequenced [15]. Here, we have isolated the full-length sequence of *SmChs1* and re-analyzed the sequence of *SmChs2* by cloning and sequencing the genes from mycelial cDNA (sequences submitted to the GenBank database under accession numbers GQ252794 and GQ252795, respectively). Surprisingly the *SmChs2* sequence determined earlier from genomic DNA [15] differed in multiple places compared to our new sequence, which caused a shift in the reading frame. Consequently, the previously reported amino acid sequence [15] was incorrect at the N-terminus of the protein; in addition, the stop codon had been wrongly predicted.

In order to determine the total number of *Chs* genes in *S. monoica*, Southern blot analyses were performed using 3 different restriction enzymes (*Sac*I, *Cla*I and *Pst*I) and 2 different probes (see Materials and Methods and Table S1 in Supplementary Material). The blots performed on the enzymatic digests obtained with *Sac*I using either of the 2 probes revealed 3 bands of different apparent molecular weights (Figure S1 in Supplementary Material). Two of the bands (ca 1 and 3 kb) systematically exhibited a higher intensity and were easily detectable, while the third band (ca 1.2 kb) was much fainter, even when using alternative sensitive detection methods (^32^P-labelled probes and autoradiography, or biotinylated probes and chemiluminescent detection) (Figure S1). This third band was never visible on any of the blots performed on the *Cla*I and *Pst*I digests (not shown), suggesting that its occurrence in the blots performed on the *Sac*I digests may have arisen from nonspecific hybridization. This is further supported by the fact that all PCR approaches used to isolate the *Chs* genes from *S. monoica* systematically generated fragments corresponding to either *SmChs1* or *SmChs2*. In addition, previous work based on chromosomal localization and Southern blot analyses suggested the occurrence of 2 *Chs* genes only in *S. monoica* [15]. Altogether, our data and earlier investigations [15] point toward the existence of a total of 2 *Chs* genes in *S. monoica.*

### Sequence analysis of SmCHS1 and SmCHS2

**Figure 1:**
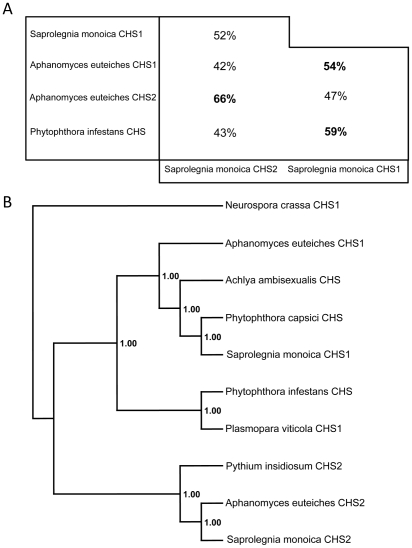
Sequence identities and phylogenetic analysis of oomycete CHS proteins. (A) Amino acid identity between the sequences of the conserved domains of SmCHS1 and SmCHS2, and the similar segments from other putative oomycete CHS proteins. The percentage of amino acid identity was calculated following alignments of the 245–744 amino acid portion of SmCHS1 and the 291–794 amino acid segment of SmCHS2. (B) Majority rule consensus tree of oomycete CHS proteins rooted with the sequence of CHS1 from *Neurospora crassa*. Numbers refer to posterior probabilities. Accession numbers are listed in Materials and Methods.

*SmChs1* codes for a polypeptide of 910 amino acids with a theoretical mass and pI of 103,100.95 Da and 8.41, respectively, while *SmChs2* encodes a protein of 962 amino acids, corresponding to a theoretical mass of 107,220.75 Da and a pI of 6.33. Detailed analysis of the SmCHS1 and SmCHS2 sequences revealed that both proteins contain the yeast and fungal conserved CHS motifs (motifs a to h, Figure S2 in Supplementary Material), including the catalytic pfam 03142 subdomain [18], [19]. The pentapeptide forming the g motif (Q(R/G)RRW) is a signature of all known *N*-acetylglucosaminyltransferases with processive activity [18], [19] and it also occurs more generally in the form QXXRW in most processive glycosyltransferases from family 2 [20]. Motifs d and e are similar to those found in fungal CHS proteins from division 1 [18], which further supports the phylogenetic relationship between oomycete and fungal chitin synthases from division 1 [12], [18]. The full-length amino acid sequences of SmCHS1 and SmCHS2 (Figure S2) share 42% similarity with each other, while 52% amino acid identity was observed when comparing the conserved segments between motifs a and h. These regions exhibited 43–66% identity with the predicted sequences from other oomycete CHS proteins (Figure 1A). The alignments revealed that SmCHS2 exhibits greater similarity with AeCHS2 than with SmCHS1, and SmCHS1 is more similar to its orthologue in *A. euteiches* than to SmCHS2 (Figure 1A). Thus, as for fungi, oomycete orthologous CHS proteins exhibit a higher similarity with each other than with their paralogues [12]. Phylogenetic analysis showed that SmCHS1 and SmCHS2 group into the 2 clusters designated previously as “CHS1” and “CHS2” [12]. However, the *P. capsici* protein groups together with SmCHS1 and not with the *P. infestans* CHS (Figure 1B), providing further evidence that oomycetes CHSs share a common ancestor that duplicated before the segregation of the orders Saprolegniales and Peronosporales.

Interestingly, the N-terminal ends of SmCHS1 and SmCHS2 exhibit high scores with the pfam 04212 domain, which corresponds to a so-called microtubule interacting and trafficking (MIT) domain [21] (Figure S2). MIT domains occur in numerous proteins having a wide range of functions [22], but their presence in carbohydrate synthases has never been described. Secondary structure prediction suggests an organization of the SmCHS1 and SmCHS2 MIT domains in 3 consecutive α-helices (Figure S3 in Supplementary Material), consistent with the typical asymmetric three-helix bundle structure experimentally determined for other MIT domains [23]. The overall topologies of SmCHS1 and SmCHS2 were predicted from hydropathy analyses (Figure S3). Both proteins exhibit a hydrophilic N-terminal end followed by a neutral segment containing the CHS consensus motifs a–h, the D,D,D,QXXRW signature of most processive glysosyltransferases [20], and the MIT domain (Figure S3). The proteins are predicted to be anchored in the plasma membrane through 7 C-terminal transmembrane domains. Searches for post-translational modifications revealed the presence of multiple potential phosphorylation sites in both proteins as well as 2 potential *N*-glycosylation sites for CHS1 but none for CHS2 (Figure S2 in Supplementary Material).

### Functional characterization of the chitin synthase genes

**Figure 2:**
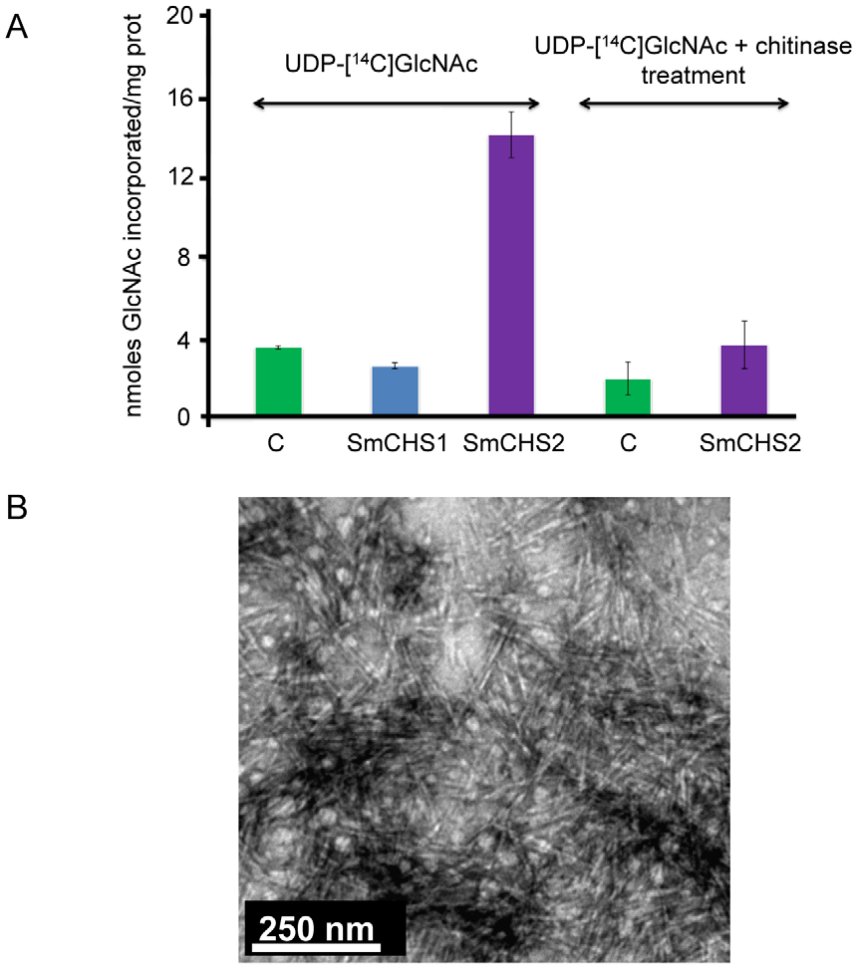
*In vitro* synthesis of chitin by SmCHS1 and SmCHS2 expressed in *Pichia pastoris*. (A) Chitin synthase activities in detergent extracts of membranes from *P. pastoris* expressing SmCHS1 and SmCHS2. Chitin synthase activity was assayed in the presence of ^14^C-labelled UDP-GlcNAc, using CHAPS extracts prepared from the membrane fractions of yeast cells expressing SmCHS1 (blue bar) or SmCHS2 (purple bars) as a source of enzyme. A control was performed in the same conditions using a CHAPS extract from untransformed *P. pastoris* cells (green bars). The radioactive polymer synthesized *in vitro* was shown to correspond to chitin by measuring its sensitivity to the action of a specific chitinase from *S. marcescens.* Standard deviations from 3 independent experiments in which each assay was performed in triplicate are shown in the Figure. (B) Electron micrograph showing crystallites of chitin synthesized *in vitro* by the recombinant SmCHS2 solubilized with CHAPS (negative staining using 2% uranyl acetate). A control performed in the same conditions using a CHAPS extract of membranes from untransformed *Pichia* cells revealed the absence of the type of crystallites visible in the micrograph.

In order to confirm that *SmChs1* and *SmChs2* encode chitin synthases, both proteins were expressed in *Pichia pastoris* with the eGFP marker at their C-termini. Chitin synthase activity was measured *in vitro* using CHAPS extracts of membranes from the recombinant zeocin-resistant yeast cells as a source of enzyme. CHAPS-solubilized microsomal fractions from the cells expressing SmCHS2 showed a 4-fold increased CHS activity compared to the control prepared from wild-type cells (Figure 2A). The lower activity observed with the control sample is attributable to chitin synthase from *Pichia*. The detergent extracts containing the recombinant SmCHS2 protein synthesized crystallites of chitin *in vitro* (Figure 2B) that were not detected when using similar preparations from the control wild-type strain (not shown). The radioactive products and the chitin crystallites synthesized *in vitro* by the detergent extracts containing SmCHS2 (Figure 2) were sensitive to chitinase digestion, thereby confirming the function of the gene and the catalytic activity of the corresponding protein. CHAPS-extracts of membranes isolated from the cells expressing SmCHS1 did not exhibit a higher level of activity compared to the control, which suggests that SmCHS1 is not responsible for the polymerization of chitin chains, as opposed to SmCHS2 (Figure 2A).

### Effect of nikkomycin Z on chitin synthase activity, mycelial growth and chitin content, and expression of *SmChs* genes

Nikkomycin Z is a well-known inhibitor of yeast and fungal chitin synthases [24], but its effect on enzymes and mycelial growth in oomycete has not been investigated. Here, we have first tested the effect of nikkomycin Z *in vitro*, using total membranes of *S. monoica* mycelium or a CHAPS extract from *Pichia* cells expressing SmCHS2 as a source of enzyme. In both cases, the drug caused a decrease in chitin synthase activity in a concentration-dependent manner, and enzyme kinetics revealed a competitive type of inhibition, with an apparent inhibition constant (Ki_app_) of 4.6+0.4 µM (Figure S4 in Supplementary Material). The apparent Km with respect to UDP-*N*-acetylglucosamine was 150+12 µM.

**Figure 3:**
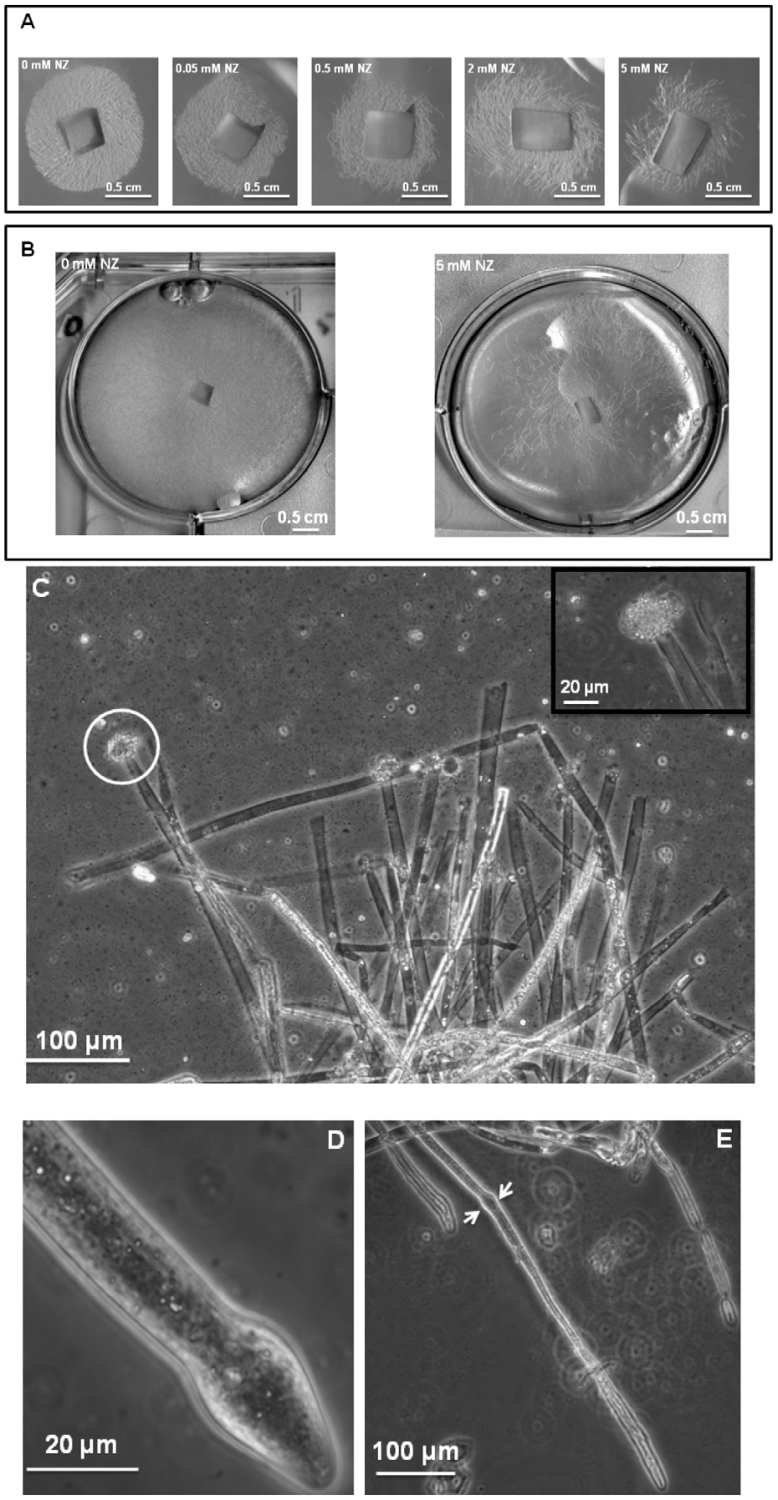
Effect of nikkomycin Z on the growth of *S. monoica* mycelium. (A) Colony growth at different concentrations of nikkomycin Z (NZ) after 2 days of culture on PDA medium. (B) As in (A) but after 5 days of culture. (C) Microscopic observations of *S. monoica* mycelium grown in the presence of 50 µM nikkomycin Z showing cell death by tip bursting (the insert shows a magnification of a bursting tip). (D) Close view of a swollen tip. (E) Observation of the same cell as in (D) but after one additional hour of growth. A deformation of the cell wall resulting from the initial tip swelling remained in this surviving cell (arrows).

Cultures of *S. monoica* hyphae in the presence of 50 µM nikkomycin Z showed that about 50% of the cells exhibited abnormal morphologies within 6 h of growth. The effect was more pronounced when using 200 µM of the inhibitor, with up to 80% abnormal cells after 6 h of culture. The morphological abnormalities consisted essentially of swollen and/or distorted hyphae (Figure S5 in Supplementary Material), reflecting an alteration of the growth most likely due to the perturbation of cell wall biosynthesis. About 50% of the cells survived after 5 days of culture in the presence of 50 µM nikkomycin Z, but all exhibited morphological abnormalities. In agreement with this rate of survival, 50% less biomass was recovered than in the absence of the drug. The cells that survived contained a similar amount of chitin in their walls as the control grown in the absence of nikkomycin Z (not shown), indicating that they were able to compensate for the inhibition of chitin synthase by the drug. Above 500 µM, nikkomycin Z provoked a dramatic decrease of the cell density (Figure 3A and B) and the systematic occurrence of numerous abnormalities along every individual hyphae. In addition, scanning electron microscopy revealed that none of the cells grew within the agar medium containing nikkomycin Z, as opposed to the control performed in the absence of inhibitor (panel C in Figure S5). Instead, all cells were crawling along the surface of the solid medium, changing their direction of growth multiple times, as if they were trying to avoid contact with the culture medium containing nikkomycin Z. Similar experiments conducted on *S. parasitica* revealed a significantly higher sensitivity of the mycelium to nikkomycin Z compared to *S. monoica*, with hardly any growth observed after several days at concentrations as low as 200 µM inhibitor (not shown).

**Figure 4:**
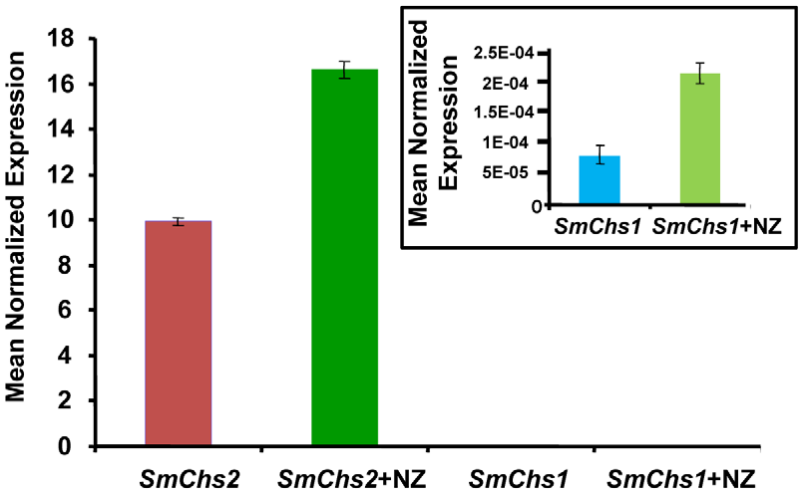
Expression analysis of *SmChs1* and *SmChs2* in *S. monoica* mycelium grown in the absence and presence of 50 µM nikkomycin Z (NZ). Quantitative RT-PCR experiments were performed on 3-day-old mycelium. The insert shows the expression levels of *SmChs1* with a different scale on the y axis. Levels of expression were normalized with the *18S rRNA* housekeeping gene and expressed as Mean Normalized Expression. Standard deviations from 3 independent sets of experiments are shown.

In order to investigate the underlying compensation mechanism in the cells that survived the presence of 50 µM nikkomycin Z, the expression of *SmChs1* and *SmChs2* was analyzed by quantitative RT-PCR using mycelium grown in the presence and absence of inhibitor (50 µM) for up to 5 days. In the absence of inhibitor, the level of expression of *SmChs2* was more than 100,000 times higher than that of *SmChs1* (Figure 4). This important difference suggests that *SmChs2* plays a major role in chitin biosynthesis in the hyphae. The presence of nikkomycin Z in the culture medium provoked an increase by 70% of the expression of *SmChs2* (Figure 4). Even though the overall expression of *SmChs1* was negligible compared to that of *SmChs2*, the expression of *SmChs1* also increased significantly (300%) in the presence of nikkomycin Z (Figure 4). These data suggest that the cells that survived the presence of nikkomycin Z did so by compensating the inhibitory effect of the drug by up-regulating the expression of the chitin synthase genes.

The mode of action of nikkomycin Z on cell death was determined by continuously observing individual cells in the presence of 50 µM inhibitor during a period of 1 h. Within a few minutes, the tip of about 50% of the cells swelled and burst, leading to cell death through the release of the cellular content in the extracellular medium (Figure 3C). This indicates that the cell wall was more fragile at the tip of these hyphae than in the absence of nikkomycin Z, most likely due to a lack or lower amount of chitin. The data suggest that chitin, which represents less than 0.5% of the total cell wall carbohydrates [11] is primarily synthesized at the tip of the hyphae where the nikkomycin-Z-sensitive chitin synthase is most active. The tips of the cells that survived also swelled and the change of shape of the cell wall remained as the hyphae elongated (Figure 3, D and E). In these surviving cells, the compensation of the chitin default occurred through overexpression of chitin synthase, as suggested above with the demonstration of the overexpression of *SmChs1* and *SmChs2* in the presence of nikkomycin Z.

## Discussion

In our early work on oomycete chitin synthases, we preliminarily reported that the chitin synthase inhibitor polyoxin D has a negative effect on the growth of *S. monoica* [11]. Thus, enzymes involved in the biosynthesis of minor cell wall carbohydrates such as chitin synthases may play important roles in cell integrity and represent potential targets for growth inhibitors of oomycetes, in addition to enzymes that form major cell wall components. We have now demonstrated this hypothesis by using *S. monoica* as a model species and showing that the more economically important pathogen *S. parasitica* exhibits an even higher sensitivity to the chitin synthase inhibitor nikkomycin Z. We also show for the first time that the *Saprolegnia* chitin synthases are primarily located at the apex of the mycelium and involved in tip growth.

Several oomycete genes are considered to encode chitin synthases owing to their sequence similarity with fungal and yeast chitin synthase genes [13]–[15]. However, their function has never been experimentally demonstrated. Here we provide the first evidence that the protein SmCHS2 from *S. monoica* indeed catalyses the formation of chitin. The protein expressed in *P. pastoris* as a GFP fusion was correctly targeted to the plasma membrane (data not shown) and was isolated in a catalytically active form after solubilization from the yeast membranes. The extracted protein was able to incorporate radioactive GlcNAc from ^14^C––labeled UDP-GlcNAc into chitin crystallites that were sensitive to the action of a specific chitinase. There is so far only one example where the heterologous expression of a chitin synthase has been reported, but the data were obtained in a non-oomycete species, the yeast *Saccharomyces cerevisiae* [16]. In general, such experiments have proven to be extremely challenging due to the occurrence of multiple transmembrane domains in chitin synthases. In addition to providing the first direct evidence for the catalytic activity of an oomycete chitin synthase, we confirm that the enzyme is inhibited by nikkomycin Z in a competitive manner. The Ki_app_ value is in the same range as previously reported for different yeast and fungal crude chitin synthase preparations while the apparent Km is 3–10 times lower [25]–[27].

Despite the occurrence in SmCHS1 of the same typical conserved chitin synthase motifs as in SmCHS2 and other CHS from division I [12], [19], no activity was measured *in vitro* when the protein was expressed in the same yeast system as SmCHS2. PCR experiments and observation by confocal microscopy of the recombinant GFP fusion protein confirmed that it was expressed and correctly targeted to the plasma membrane (not shown). If we assume that the enzyme was correctly folded, these data suggest that, as opposed to SmCHS2, SmCHS1 is not involved in the polymerization of insoluble chains of chitin. Interestingly *A. euteiches* has been shown to synthesize rather abundant (10% of cell wall content) GlcNAc-based carbohydrates that are water-soluble and exposed at the surface of the cells [12]. These carbohydrates were clearly different from chitin and, due to their solubility in water, it was concluded that they correspond to heterosaccharides that are possibly branched [12]. Since the cell wall of *A. euteiches* is devoid of chitin, it was proposed that the *AeChs1* and *AeChs2* genes encode proteins that do not form chitin but that are involved in the formation of the soluble GlcNAc-based heterosaccharides [12]. However, the actual *N*-acetylglucosaminyltransferase activity of AeCHS1 and AeCHS2, if any, remains to be demonstrated. By analogy with the *Aphanomyces* proteins, it is possible that SmCHS1 is involved in the biosynthesis of GlcNAc-based carbohydrates different from chitin. This hypothesis would concur with the inability of the recombinant protein to form chitin *in vitro*, as shown here. Alternatively, SmCHS1 may be involved in chitin biosynthesis during non-mycelial developmental stages, which would be consistent with the observed low level of expression in the mycelium. It is noteworthy that the assay used here was optimized for the measurement of GlcNAc incorporation into insoluble chitin polymers or into chito-oligosaccharides of a degree of polymerization higher than 6 [11], [17]. Thus, another possibility explaining the absence of *in vitro* activity with the recombinant SmCHS1 is that the protein may form chito-oligosaccharides of a low degree of polymerization that could possibly be involved in priming chitin biosynthesis.

Our data based on the use of nikkomycin Z strongly suggest that chitin synthase activity is essentially located at the hyphal tip in *Saprolegnia*. Indeed, inhibition of chitin biosynthesis led to the formation of a weaker wall at the apex of the hyphae, which was accompanied by a swelling of the cell primarily at its tip, followed by cell lysis. From these observations, and similar to the endosome-to-plasma membrane pathway described in yeast for membrane proteins [28], it is tempting to speculate that the MIT domains of SmCHS1 and SmCHS2 are involved in the apical delivery of chitin synthase via a direct or indirect interaction with the cytosleleton, and/or in the recycling of the enzyme through endosomal membranes. Membrane microdomains similar to lipid rafts have been shown to be involved in cell polarization in fungi [29]. This is consistent with our recent finding that the *Saprolegnia* chitin synthase activity is associated to such membrane structures [30], and with the present observation that chitin synthase is primarily located at the apex of the cells.

To date the CHS proteins from *S. monoica* represent the only examples of processive carbohydrate synthases that contain MIT domains. Similarly to SmCHS1 and SmCHS2, some of the *Saprolegnia* [31] and *Phytophthora* [32] cellulose synthases contain domains that seem to be specific to the oomycete enzymes. These domains belong to the Pleckstrin Homology (PH) family of domains [31], [32]. PH domains occur in a wide range of proteins involved in intracellular signaling or as constituents of the cytoskeleton [33]. They have been associated to different functions, especially the targeting of proteins to appropriate cellular compartment [33]. Altogether, these observations suggest that oomycete carbohydrate synthases have evolved by acquiring specific domains at their N-terminal ends that guide their cellular trafficking and membrane targeting.

As mentioned above, our data concur with a localization of chitin synthase activity at the apex of *Saprolegnia* hyphae, which implies a role in tip growth. This represents the first evidence of the biological function of chitin synthases in oomycetes. The data suggest that chitin is essentially synthesized at the tip of the hyphae, consistent with the previous observation that chitin is a minor cell wall component of *S. monoica* mycelial walls (<0.5% of the total cell wall carbohydrates) [11]. The chitin granules that have been shown to occur in all parts of the mycelial cell wall [11] most likely arise primarily from the apical site of synthesis and, as the cells grow, get distributed along the older parts of the hyphal walls. Interestingly, ultrastructural studies of the cell wall of the closely related species *Achlya bisexualis* have shown that cellulose is mainly located in the subapical and older parts of the hyphae, while the tip of the cells are devoid of cellulose and consist essentially of β-(1→3)-glucan [34], [35]. Although no similar studies have been performed in *Saprolegnia*, *Saprolegnia* cells most likely exhibit a similar distribution of cellulose and β-(1→3)-glucan in their walls as *Achlya*. In this case, chitin would substitute for cellulose at the tip of the hyphae to maintain a sufficient mechanical support to the apical wall. Indeed, the type of crystalline chitin that occurs in *Saprolegnia* is similar to fungal chitin and exhibits comparable physical and mechanical properties as cellulose [11]. A matrix that would consist essentially of amorphous β-(1→3)-glucan is not expected to be able to provide sufficient mechanical support to the cell and needs to be reinforced by crystalline polymers such as chitin or cellulose [6], [7]. From these observations, we reasoned that despite its low abundance in *Saprolegnia* cell walls, chitin plays an important role in tip growth and stabilization of the apical wall, and thus represents an interesting target of anti-oomycete drugs. This was verified by confirming that nikkomycin Z was indeed able to inhibit the mycelial chitin synthase activity and provoked cell lysis through tip bursting. Interestingly, the mycelium from *S. parasitica* showed an increased sensitivity to the inhibitor compared to *S. monoica*, which confirms the potential of using chitin synthase as a target for controlling the diseases provoked by devastating pathogenic oomycetes. The nikkomycin Z concentrations required for inhibiting the growth of *S. parasitica* were in the range 200 µM (data not shown). The mycelium from *S. monoica* could overcome such concentrations, with about 50% cell death, but all surviving cells grew slower and exhibited multiple abnormalities compared to the untreated control. The morphological alterations were similar to those reported earlier when growing the mycelium in the presence of Congo Red [36], which interferes with both chitin and cellulose formation. The cells surviving the nikkomycin Z treatment exhibited a higher level of expression of *SmChs1* and *SmChs2*, most likely to compensate for the inhibitory effect of the drug.

In conclusion, due to the rather high concentrations of nikkomycin required to provoke cell lysis, or at least inhibit the growth of the mycelium, this drug may not be usable in practice to control *Saprolegnia* infections of fish. It remains however that chitin synthase represents one of the potential targets of anti-*Saprolegnia* drugs. The development of drugs with a higher affinity for chitin synthases than nikkomycin Z should allow the use of lower concentrations of inhibitors and be thus friendlier to the environment. Combining such chitin synthase inhibitors with compounds that are directed toward other cell wall synthesizing enzymes or other key biochemical pathways represents an interesting strategy to control the diseases caused by *Saprolegnia*.

## Materials and Methods

### Strain and culture conditions

The strains *S. monoica* Pringsheim 53–967 Dick and *S. parasitica* Coker were obtained from the Centraal Bureau voor Schimmel Culture (CBS, Baarn, The Netherlands) and maintained on Potato Dextrose Agar (PDA). The mycelium used for all experiments was grown in the liquid medium of Machlis [37] for 3–5 days at 25°C, as previously described [11].

### RNA extraction, RACE and qRT-PCR

Total RNA was extracted and purified from 100 mg of 3-days old *S. monoica* mycelium, using the RNeasy Plant Mini Kit (Qiagen) and an on-column DNaseI digestion. Reverse transcription experiments were performed with 4.5 µg total RNA using the Superscript III First Strand cDNA Synthesis kit (Invitrogen) following the manufacturer's instructions. For the PCR amplification of *SmChs2*, 1 µl of the synthesized cDNA was used with primers (*Chs2*Fwd and *Chs2*Rev, Table S1 in Supplementary Material) designed from the putative *SmChs2* gene sequence isolated previously (GenBank accession number U19946). Similarly, *Chs1*Fwd and *Chs1*Rev primers (Table S1) were designed from the partial sequence of the putative *Chs1* gene from *P. capsici* (GenBank accession number U42304) and used for the amplification of a conserved region of *Chs1*. All PCR reactions were performed using Phusion High-Fidelity DNA Polymerase (Finnzymes), according to the manufacturer's instructions. An RNA ligase-mediated RACE kit (RLM-RACE kit, Ambion) was used to isolate the 5′ and 3′ ends of *SmChs1* and amplify the sequence of the 3′ end of *SmChs2* for verifying its sequence. The following sets of nested reverse primers were used to obtain the 5′ end of *SmChs1*: *Chs1*Rev1 and C*hs1*Rev2 (Table S1). The 3′ end of the gene was isolated using the nested forward primers *Chs1*Fwd1 and *Chs1*Fwd2 (Table S1), while the 3′ end of *SmChs2* was amplified with the primers *Chs2*Fwd1 and *Chs2*Fwd2 (Table S1). The full-length *SmChs1* and *SmChs2* genes were amplified from mycelial cDNA to confirm their complete sequences. The following primers were used for this purpose: *Chs1*FLFwd and C*hs1*FLRev for the amplification of *SmChs1*, and *Chs2*Fwd and *Chs2*FLRev for the amplification of *SmChs2* (Table S1). The full-length genes were cloned into the pENTR-D-TOPO vector (Invitrogen) using the Gateway technology, according to the manufacturer's instructions. The constructs were transformed into One Shot TOP10 *Escherichia coli* cells that were chemically competent (Invitrogen) and their sequenced confirmed (MWG, Germany).

Quantitative Real-Time PCR (qRT-PCR) analyses were normalized against the *18S rRNA* [31], ubiquitin and glyceraldehyde-3-phosphate dehydrogenase (GAPDH) genes from *S. monoica*. Since the sequences of the latter 2 genes were unknown, we first amplified them by designing primers from the *S. parasitica* ATCC90214 mycelial sequence library (www.oomycete.org). *Ub*Fwd and *Ub*Rev were used to amplify the ubiquitin gene, while *GAPDH*Fwd and *GAPDH*Rev were the primers used for isolating the *GAPDH* gene (Table S1). The PCR products were cloned into pUC19 using the *Sma*I site and the constructs were transformed into *E. coli* competent cells as above. For qRT-PCR experiments, total RNA was extracted from the mycelium grown in the absence or in the presence of 50 µM nikkomycin Z for either 3 days or 5 days. One µg RNA was converted into cDNA using the SuperScript III First-Strand Synthesis SuperMix kit for qRT-PCR (Invitrogen). The cDNA was diluted to 2.4 ng/µl, and 2.5 µl of the diluted cDNA was used as a template for qRT-PCR analysis. The sequences of the primers are presented in Table S2 (Supplementary Material). The cDNA was amplified using the iQ SYBR Green Supermix (Bio-Rad) on a Bio-Rad iCycler. The reactions were performed in triplicates and repeated on two independent biological samples. The PCR conditions consisted of an initial denaturation at 95° for 3 minutes, followed by 60 cycles of denaturation at 95° for 30 seconds, annealing at 58° for 30 seconds and elongation at 72° for 30 seconds. A dissociation kinetics analysis was performed at the end of the experiment to check the specificity of the annealing. Results were analysed with the Q-gene software [38]. Identical results were obtained with all 3 housekeeping genes used. Only the normalized results obtained with the *18S rRNA* gene are presented.

### Southern blot analysis

DNA from *S. monoica* mycelium grown for 3 days in the liquid culture of Machlis [37] was extracted in the presence of cetyltrimethylammonium bromide [39]. The genomic DNA was resuspended in TE buffer (10 mM Tris-HCl pH 8.5 containing 0.1 mM EDTA) and 100 µg was digested with *Sac*I, *Cla*I or *Pst*I (Fermentas) at 37°C overnight. Aliquots of the digests containing the restriction fragments were separated by electrophoresis on a 0.7% (w/v) agarose gel. After depurination and alkaline denaturation, the DNA fragments were transferred to a positive nylon membrane (Hybond N+, Amersham) and subjected to Southern hybridization [40]. The probes corresponding to conserved amino acid sequences were prepared using the NEBlot Phototope kit (New England BioLabs) after PCR amplification of a plasmid DNA template containing *SmChs2*, using the primer pairs *Chs2*Probe1Fwd/C*hs2*Probe1Rev and *Chs2*Probe2Fwd/*Chs2*Probe2Rev (Table S1). The probes corresponding to the 1404–1704 nucleotide segment of *SmChs2* (Probe 1) and 876–1233 nucleotide sequence of *SmChs2* (Probe 2) were amplified using the Phusion High-Fidelity DNA Polymerase from Finnzymes. They were labeled with biotin and ^32^P in the presence of [α-^32^P]dCTP (Perkin Elmer, Boston, MA; Megaprime DNA Labeling Systems, Amersham Biosciences, Uppsala, Sweden) according to the manufacturer's instructions. Hybridization was performed overnight at 65°C in the presence of 20 ng/ml of each probe. The detection of the signals was performed using the chemiluminescent Phototope Star detection kit following the manufacturer's instructions (New England BioLabs), and a Biorad CCD camera.

### Bioinformatics and phylogenetic analyses

All sequences were edited using the software BioEdit [41] and assembled from an initial database of oomycete chitin synthases. Alignments were performed using ClustalW [42]. The SmCHS1 and SmCHS2 full-length sequences as well as the MIT domain sequences alone were used to perform a BLASTp search against non-redundant protein databases from the National Centre for Biotechnology (http://www.ncbi.nlm.nih.gov). The full-length sequences were also used for similarity searches against the *P. infestans* database at the Broad Institute (http://www.broadinstitute.org/annotation/genome/phytophthora_infestans/MultiHome.html). The resulting oomycete dataset consisted of the putative chitin synthases (accession numbers in parentheses) from *P. infestans* (PITG_02050.1), *A. euteiches* (EU522489 and EU447431 for AeCHS1 and AeCHS2 respectively), *Plasmopara viticola* (AY05254), *Pythium insidiosum* (DQ116421), *P. capsici* (U42304), and *Achlya ambisexualis* (U55044). The phylogenetic tree was rooted with the sequence of CHS1 from *Neurospora crassa* (accession number M73437). The dataset of MIT-containing proteins was prepared by gathering the MIT domain sequences (accession numbers in parentheses) from *Mus musculus* (1WFD_A), the *Aspergillus oryzae* calpain-like protease PalBory (AB020321), the vacuolar sorting protein VPS4 from *Entamoeba histolytica* (XM_649013), the *Sulfolobus solfataricus* AAA ATPase family protein (NP_342401), the *Homo sapiens* sorting nexin 15 (AAH14520), and the *Theileria annulata* putative AAA family ATPase (XM_949947).

The phylogenetic analyses of the protein datasets were performed with the software MrBayes (v3.1; [43]) using a Bayesian inference [44] under the model suggested by the program ProtTest (http://darwin.uvigo.es/software/prottest_server.html; [45]), which estimates likelihood scores under different models. The WAG+G+F amino acid substitution model was suggested and implemented in the Bayesian inference [46]. The Bayesian analyses consisted of two Markov chains run in parallel. Each of them was flanked to other three simultaneous “heated” chains (Metropolis coupled Markov chain Monte Carlo; [44]) to avoid getting trapped in a local maximum. The chains were run for 500,000 generations, with sampling only once every 100 generations and collecting at the end 5,000 trees. In order to avoid early pre-convergence trees, the first 25% (burning) trees were discarded. The majority rule consensus tree was calculated on the remaining trees, with posterior probability on every node as a measure of confidence of the analyses.

Transmembrane domains were predicted using the TMHMM [47] program at http://www.cbs.dtu.dk/services/TMHMM-2.0/and Kyte and Doolittle hydropathy plots [48] were obtained using the tool available at http://fasta.bioch.virginia.edu/fasta_www2/fasta_www.cgi. Potential phosphorylation and glycosylation sites were identified using the NetPhos 2.0 server (http://www.cbs.dtu.dk/services/NetPhos/) and the NetNGlyc 1.0 server (http://www.cbs.dtu.dk/services/NetNGlyc/), respectively. The search for putative domains in SmCHS1 and SmCHS2 was performed in the Pfam motif database using MotifScan [49] (http://hits.isb-sib.ch/cgi-bin/PFSCAN). The diagrams representing the predicted topology of SmCHS1 and SmCHS2 were drawn using the TOPO2 program at http://www.sacs.ucsf.edu/TOPO-run/wtopo.pl.

### Treatment with nikkomycin Z and optical microscopy

The effect of the chitin synthase inhibitor nikkomycin Z on the growth of *S. monoica* and *S. parasitica* was tested on cultures of mycelium on PDA plates supplemented with 0, 25, 50, 200, 500, 2000 or 5000 µM nikkomycin Z (Sigma) in either 90-mm Petri plates or 6-well culture plates (Falcon). The microscopic observations were performed on the mycelium grown in 20 ml of liquid medium [37] supplemented with the indicated concentrations of nikkomycin Z. Aliquots of the control and nikkomycin-treated cultures were aseptically removed at different times and mounted on microscope slides. Observations were performed using an Olympus BX-51 optical microscope fitted with a camera.

### Field Emission-Scanning Electron Microscopy (FE-SEM)

The mycelium from the control and nikkomycin-treated cultures (5 mM inhibitor) grown on PDA was flash-frozen in liquid nitrogen, freeze-dried and observed by FE-SEM. The samples were mounted onto a metal substrate using carbon tape, and coated with a thin layer of Au–Pd. A Hitachi S-4800 scanning electron microscope operated at 1 kV was used to capture secondary electron images of the surfaces of the samples.

### Preparation of microsomal fractions from *S. monoica* mycelium, chitin synthase assay and enzyme kinetics

*S. monoica* was grown in 140-mm Petri plates for 3 days in 100 ml liquid medium (Machlis) [37] inoculated with about 30 agar plugs of 5 mm cut from cultures grown on PDA. The mycelium was collected, washed extensively with water and dried under vacuum on filter paper. All the subsequent steps were performed at 4°C. The cells were homogenized in an extraction buffer (Tris-HCl 10 mM pH 7.4) containing a plant protease inhibitor cocktail (Sigma) using a Waring blender. The homogenate was centrifuged at 5,000 g for 10 minutes. The supernatant was filtered through two layers of Miracloth and centrifuged at 100,000 g for 1 hour (Beckman-Coulter Optima L-100 XP ultracentrifuge). The pelleted membranes were resuspended in extraction buffer and used for protein [50] and chitin synthase assays.

Chitin synthase activity was measured as described earlier [11], [17]. Briefly, 50 µl of enzyme preparation was mixed with 150 µl of reaction buffer consisting of 10 mM Tris-HCl pH 7.4, 10 mM MgCl_2_, 20 mM *N*-acetyl-d-glucosamine, 1.25 µg trypsin/ml, 0.5 mM UDP-*N*-acetyl-d-glucosamine and 434 nM UDP-*N*-acetyl-d-[U-^14^C]glucosamine (318 mCi/mmol; Amersham). The reaction was incubated for 1 hour at room temperature and stopped with 400 µl of ethanol. After an overnight precipitation at −20°C, insoluble polysaccharides were recovered on Whatman GF/C glass-fiber filters and subsequently washed with water and 70% ethanol. The radioactivity retained on the filters was detected by liquid scintillation (Packard 1500 Tri-Carb). The determination of the apparent Km of chitin synthase was performed in the assay conditions described above, but by using different UDP-GlcNAc concentrations (0.1, 0.5, 1 and 2 mM). The apparent Ki of the enzyme for nikkomycin Z was obtained from Dixon plots by performing kinetics in the presence of varying concentrations of nikkomycin Z (0, 0.2, 1, 5, 15 µM) and UDP-GlcNAc (0.1, 0.5 and 1 mM) over a period of 30 min reaction.

### Determination of mycelial chitin content

The chitin content of mycelial cells was measured by assaying the amount of glucosamine released by acid hydrolysis of the wall-bound chitin [51]. Cell wall samples were prepared from about 1 g fresh weight of mycelium by grinding the cells in liquid nitrogen and resuspending the homogenate in 2 ml deionized water. After centrifugation at 13,000 g for 10 minutes at 4°C, the pelleted material was freeze-dried overnight. Known amounts of the samples (2 to 6 mg) were hydrolyzed with 1 ml of 6 M HCl at 100°C for 4 h. After cooling to room temperature, the hydrolysate (0.2 ml) was added to 0.25 ml of 4% acetylacetone in 1.25 M sodium carbonate and heated for 1 h at 90°C. After cooling, 2 mL of ethanol were added under agitation to dissolve the precipitate. Finally, 0.25 ml of Ehrlich reagent (1.6 g of *N*,*N*-dimethyl-p-aminobenzaldehyde in 60 mL of a 1∶1 mixture of ethanol and concentrated HCl) was added and the absorbance was measured at 530 nm. The chitin content, expressed as micrograms of glucosamine hydrochloride per mg of cell wall (dry weight) was calculated from a standard curve prepared with known amounts of glucosamine hydrochloride.

### Expression of SmCHS1 and SmCHS2 in *Pichia pastoris*

SmCHS1 and SmCHS2 were expressed as fusion proteins with eGFP at their C-terminus. For this purpose, the corresponding genes were cloned in the pDONR221 vector using the BP clonase kit (Invitrogen) and the primers *Chs1Pichia*Fwd/*Chs1Pichia*Rev and *eGfp*Fwd*Chs*1/*eGfp*Rev*Chs*1 for *SmChs1*, and *Chs2Pichia*Fwd/*Chs2Pichia*Rev and *eGfp*Fwd*Chs*2/e*Gfp*Rev*Chs*2 for *SmChs2* (Table S1). The donor vectors were recombined into a Gateway compatible version of the *Pichia* vector pPICZ using the LR clonase kit (Invitrogen). Zeocin resistant colonies were screened for GFP fluorescence using a confocal microscope (LSM510, Zeiss) and the positive clones exhibiting the highest signal were used for subsequent chitin synthase assays.

### Chitin synthase assay using detergent extracts of *Pichia* membranes

The *Pichia* cells grown in the buffered glycerol complex medium (BMGY: 100 mM potassium phosphate pH 6.0, 1% yeast extract, 2% peptone, 1.34% yeast nitrogen base, 4×10^−5^% biotin, 1% glycerol) for one day at 30°C were collected by low-speed centrifugation and resuspended in the BMMY medium at a final OD_600_ of 1 (BMMY medium: as BMGY but with 0.5% methanol instead of glycerol). The cells were induced for one day at 30°C with 0.5% (v/v) methanol, harvested and resuspended in a lysis buffer (Tris-HCl 10 mM pH 7.4) supplemented with a yeast protease inhibitor cocktail (Sigma). The cells were disrupted in an Aminco French pressure cell (10,000 psi), and the microsomal fraction was obtained by differential centrifugation as described above for the isolation of the membrane fraction from the mycelium of *S. monoica*. The membrane fraction was resuspended in lysis buffer and diluted to a final concentration of 1 mg/ml protein prior to protein extraction in the presence of 0.5% 3-[(3-cholamidopropyl)dimethylammonio]-1-propanesulfonate (CHAPS) at 4°C under gentle stirring. The preparation was centrifuged at 100,000 g for 1 h (4°C) and the supernatant corresponding to the CHAPS extract was used for chitin synthase assays or enzyme kinetics as described above.

### Characterization of the chitin synthesized *in vitro* and observation by transmission electron microscopy

Product characterization was performed by enzymatic hydrolysis of the radioactive chitin synthesized *in vitro* in the presence of UDP-*N*-acetyl-d-[U-^14^C]glucosamine (see enzyme assay conditions). The ethanol-insoluble polysaccharides were pelleted by a 10 min centrifugation at 10,000 g, washed with 1 ml hydrolysis buffer (50 mM sodium acetate pH 5.0) and pelleted again. The insoluble polysaccharides were resuspended in 200 µl of hydrolysis buffer containing the specific chitinase from *Serratia marcescens* (Sigma) used at 25 µg/ml (final concentration). Triplicates were run in parallel. The samples were incubated for 48 hours at 25°C and the reaction mixtures were supplemented every 24 hours with freshly prepared chitinase. The non-hydrolyzed polysaccharides were precipitated overnight in ethanol at −20°C and recovered by filtration on glass-fiber filters. The radioactivity retained on the filters was determined by scintillation counting as described above. The extent of hydrolysis was determined by comparison with controls in which the hydrolytic enzyme had been replaced by the corresponding incubation buffer. For electron microscopy observation of the chitin synthesized *in vitro* by the recombinant chitin synthases expressed in *Pichia* and solubilized with CHAPS, a drop of the reaction mixture obtained after incubation in the same conditions as for a chitin synthase assay, but in the presence of nonradioactive UDP-GlcNAc, was deposited on carbon-coated grids and observed after negative staining with 2% uranyl acetate as described earlier [17]. Control experiments were performed by using a similar protein fraction prepared from the wild-type *Pichia* cells.

## Supporting Information

Table S1 Sequences of the primers used for the RACE PCR experiments.
(0.04 MB DOC)

Table S2 Sequences of the primers used for the quantitative RT-PCR experiments.
(0.03 MB DOC)

Figure S1 Restriction map of the *SmChs2* cDNA sequence and Southern blot analysis of *S. monoica Chs* genes. (A) Positions of the probes and restriction sites used (*P* =  *Pst*I, *S* =  *Sac*I) in the cDNA of *SmChs2*. (B) Genomic DNA was digested with *Sac*I, transferred to nylon membranes and hybridized with biotinylated probes 1 and 2 designed on conserved sequences between the 2 *SmChs* genes and corresponding to amino acid positions 468–568 and 292–411, respectively (see Materials and Methods).
(0.07 MB JPG)

Figure S2 Alignment of the amino acid sequences of SmCHS1 and SmCHS2 with the *Neurospora crassa* and *Saccharamyces cerevisiae* CHS2 sequences. The CHS conserved motifs a to h are boxed. The amino acid sequence of the MIT domain (pfam 04212) is boxed and highlighted in yellow. Ser, Thr and Tyr residues that are potentially phosphorylated are shown in bold font and underlined. Potential N-glycosylation sites were identified in SmCHS1 only. They are highlighted in blue colour and bold font. Asterisks indicate residues that are identical in all sequences analysed, colons indicate conserved substitutions between the sequences and periods indicate semi-conserved substitutions.
(0.09 MB PDF)

Figure S3 Sequence alignment of the MIT domains from SmCHS1 and SmCHS2, and predicted topology for both proteins. (A) Alignment of the MIT domains highlighting their organization into 3 α-helices. The most conserved residues of MIT domains according to Scott et al. [23] are shown below the alignment. (B) and (C), topology prediction of SmCHS1 and SmCHS2, respectively. The amino acids belonging to the MIT domain [23] and D,D,D,QXXRW conserved motif of most processive glycosyltransferases [20] are highlighted in blue and purple, respectively.
(0.55 MB JPG)

Figure S4 Enzyme kinetics performed on membrane fractions from *S. monoica* in the presence of various concentrations of UDP-GlcNAc and nikkomycin Z. The Dixon plots were used to extrapolate the values for the apparent Km and inhibition constant (K_i_) of chitin synthase activity for UDP-GlcNAc and nikkomycin Z, respectively. Identical results were obtained from both the recombinant SmCHS2 (not shown) and the membrane fractions from *S. monoica* mycelium.
(0.06 MB JPG)

Figure S5 Effect of nikkomycin Z on the morphology of *S. monoica* mycelium. (A) Mycelium grown in the absence of inhibitor. (B) Mycelium grown for 5 days in liquid medium supplemented with 50 µM nikkomycin Z. The arrows point to morphological abnormalities. Inserts show magnifications of hyphal tips. (C) FE-SEM micrographs of hyphae grown on PDA for 5 days in the absence or presence of 5 mM nikkomycin Z. The figure shows different magnifications of the hyphae grown in the presence of nikkomycin Z. The highest magnification (bottom right micrograph) shows the morphology of a bursting hyphal tip.
(0.90 MB TIF)
