# Supplementary material for: Chitin Synthases from Saprolegnia Are Involved in Tip Growth and Represent a Potential Target for Anti-Oomycete Drugs
Source: PLoS Pathog. 2010 Aug 26;6(8):e1001070. doi: 10.1371/journal.ppat.1001070 (PMC2928807; doi:10.1371/journal.ppat.1001070)
Supplement: Table S1 — Sequences of the primers used for the RACE PCR experiments. (0.04 MB DOC) [file ppat.1001070.s001.doc]

**Table S1. Sequences of the primers used for the RACE PCR experiments**.

| **Name** | **Sequence** |
| --- | --- |
| *Chs2*Fwd | CACCATGAGTGACCAGCTCGACCTCGCGGC |
| *Chs2*Rev | TGCTCTCTGCACGGGCAACCACAACCCGAC |
| *Chs1*Fwd | AATGAGGACGAGAACGAGCTCCGGTCG |
| *Chs1*Rev | AGCTTGTAAAAGGACGACTTGGTTGGC |
| *Chs1*Rev1 | TCTCCTTGGTCATTTGCAGCGAGTGTTC |
| *Chs1*Rev2 | CAGAACGTTGTTGCAAACCTTGCGGAGT |
| *Chs1*Fwd1 | TCCGGTCGACACTCCGCAAGGTTTGCAA |
| *Chs1*Fwd2 | ACTACACGGTCCTCCTCGATGTTGGGAC |
| *Chs2*Fwd1 | TGTCGGTGGCTTGATTGTCTTTGC |
| *Chs2*Fwd2 | TTTGGCTCTACGTTGTGACGGACT |
| *Chs1*FLFwd | CACCATGCCGCCCAAGCGACCGACGACCGA |
| *Chs1*FLRev | CTAGCGCATGCGGTTGTACGGCGCTTGG |
| *Chs2*FLRev | TTAGACTTGTTGGTAGGCGCCGCCGCGG |
| *Ub*Fwd | ATGCAAATCTTCGTCAAGACGCTCACC |
| *Ub*Rev | GGTCTTGCCCGTCAGCGTCTTGACGAA |
| *GAPDH*Fwd | TCAAGCTTGGCATCAACGGCTTTGGCC |
| *GAPDH*Rev | CGAGGCGTTCGAGACGACGTGCGCGGA |
| *Chs2*Probe1Fwd | GTCATTGCCGCACAGCACTTCGAGTAC |
| *Chs2*Probe1Rev | GGCGATCGCGTCCTTGACATAGTGCAT |
| *Chs2*Probe2Fwd | ATGTACAACGAAGAAGGCTCAGAGATC |
| *Chs2*Probe2Rev | TTGAAGAACCACAGATGCGAGTTGAGC |
| *Chs1Pichia*Fwd | GGGGACAAGTTTGTACAAAAAAGCAGGCTCATAATGCCGCCCAAGCGACCGACGAC |
| *Chs1Pichia*Rev | CTCCTCGCCCTTGCTCACCATGCGCATGCGGTTGTACGGCGCT |
| *eGFP*- *Chs1*Fwd | AGCGCCGTACAACCGCATGCGCATGGTGAGCAAGGGCGAGGAG |
| *eGFP*Rev | GGGGACCACTTTGTACAAGAAAGCTGGGTCTAGTGATGGTGATGATGGTGCTTGTACAGCTCGTCCATGCC |
| *Chs2Pichia*Fwd | GGGGACAAGTTTGTACAAAAAAGCAGGCTCATAATGTCTGACCAGCTCGACCTCGC |
| *Chs2Pichia*Rev | CTCCTCGCCCTTGCTCACCATGACTTGTTGGTAGGCGCCGCCG |
| *eGFP*- *Chs2*Fwd | CGGCGGCGCCTACCAACAAGTCATGGTGAGCAAGGGCGAGGAG |
