# Supplementary material for: Chitin Synthases from Saprolegnia Are Involved in Tip Growth and Represent a Potential Target for Anti-Oomycete Drugs
Source: PLoS Pathog. 2010 Aug 26;6(8):e1001070. doi: 10.1371/journal.ppat.1001070 (PMC2928807; doi:10.1371/journal.ppat.1001070)
Supplement: Table S2 — Sequences of the primers used for the quantitative RT-PCR experiments. (0.03 MB DOC) [file ppat.1001070.s002.doc]

**Table S2.** Sequences of the primers used for the quantitative RT-PCR experiments.

| **Name** | **Sequence** |
| --- | --- |
| *Chs1* | Fwd ATCGAACGCGCAATTCAAGCTGAG  Rev TCCAACACTTCCTTGCCTTTGTGC |
| *Chs2* | Fwd TTTGGCTCTACGTTGTGACGGACT  Rev TGACGTTAAAGAAGCCGACGACGA |
| *18S rRNA* | Fwd ACGAAAGTTAGGGGATCGAA  Rev CCCTTCCGTCAATTCCTTTA |
| *Ub* (Ubiquitin) | Fwd TCCAAGACAAGGAAGGTATTCCGC  Rev ATATTGTAGTCCGAGAGGGTGCG |
| *GAPDH* | Fwd AGATGGCAACTTGATCGTCGATGG  Rev AGCCTTCTCAGTGGTCGTGAAGA |
