# Supplementary figures and images for: Chitin Synthases from Saprolegnia Are Involved in Tip Growth and Represent a Potential Target for Anti-Oomycete Drugs
Source: PLoS Pathog. 2010 Aug 26;6(8):e1001070. doi: 10.1371/journal.ppat.1001070 (PMC2928807; doi:10.1371/journal.ppat.1001070)

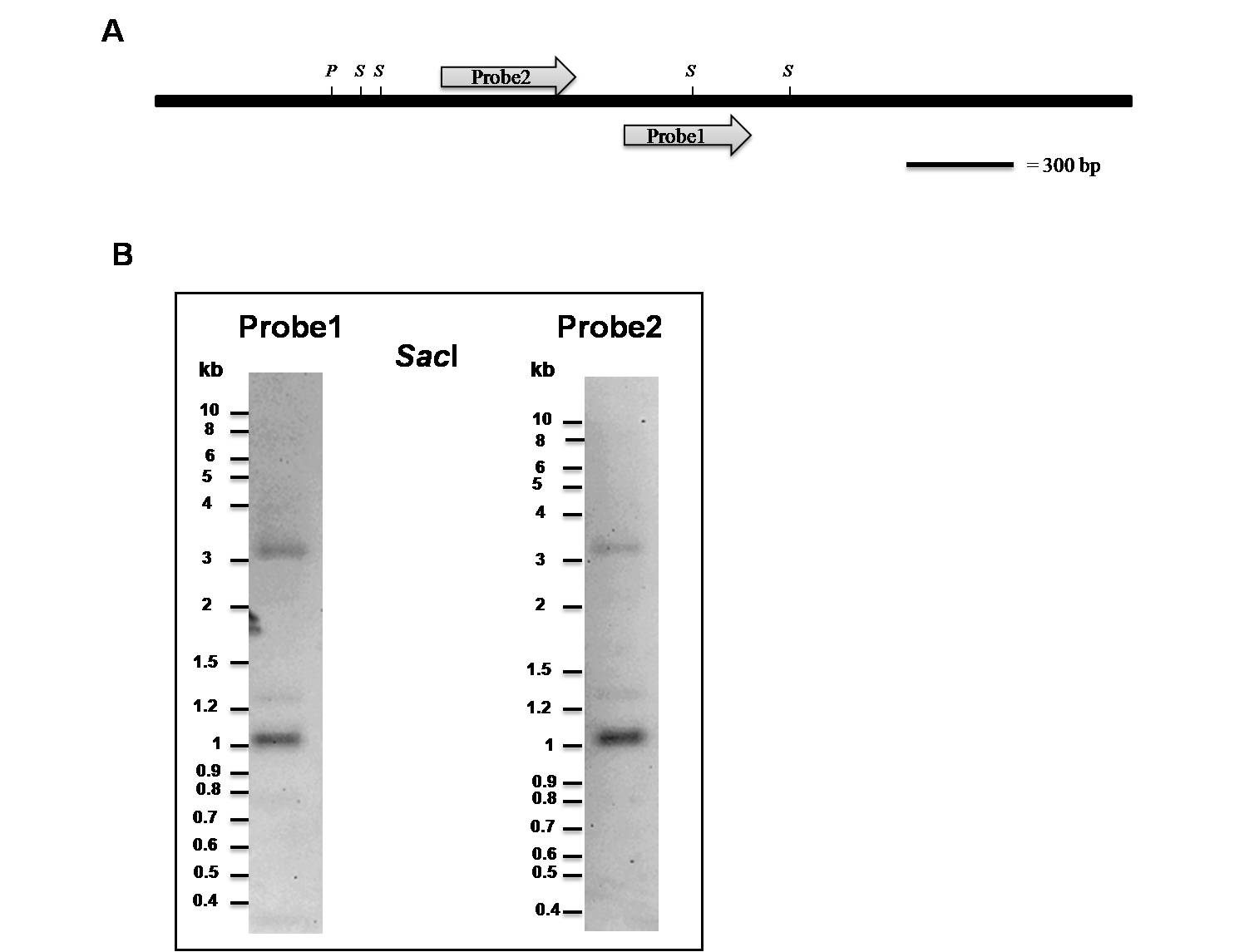

Supplement: Figure S1 — Restriction map of the SmChs2 cDNA sequence and Southern blot analysis of S. monoica Chs genes. (A) Positions of the probes and restriction sites used (P = PstI, S = SacI) in the cDNA of SmChs2. (B) Genomic DNA was digested with SacI, transferred to nylon membranes and hybridized with biotinylated probes 1 and 2 designed on conserved sequences between the 2 SmChs genes and corresponding to amino acid positions 468–568 and 292–411, respectively (see Materials and Methods). (0.07 MB JPG) [file ppat.1001070.s003.jpg]

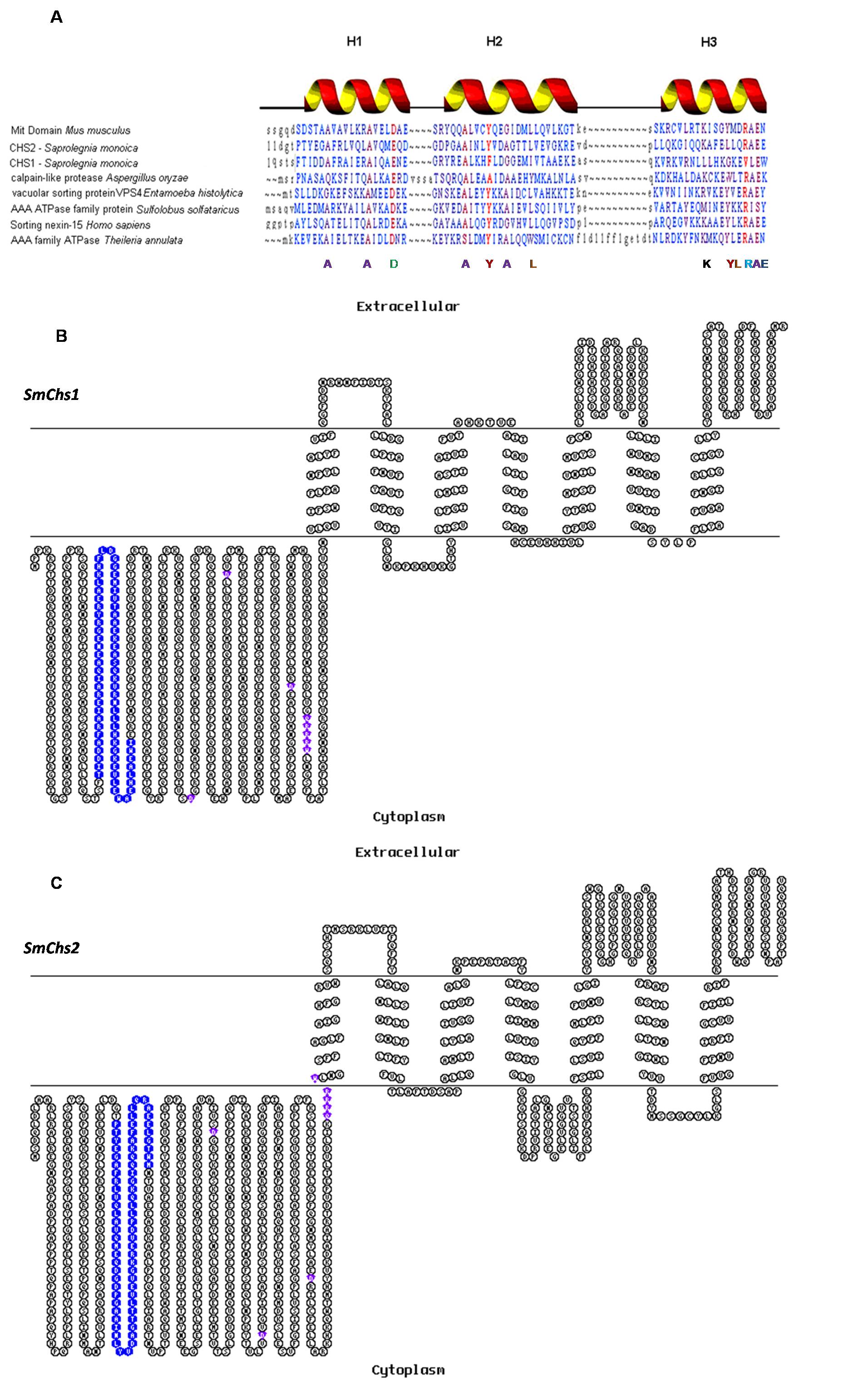

Supplement: Figure S3 — Sequence alignment of the MIT domains from SmCHS1 and SmCHS2, and predicted topology for both proteins. (A) Alignment of the MIT domains highlighting their organization into 3 α-helices. The most conserved residues of MIT domains according to Scott et al. [23] are shown below the alignment. (B) and (C), topology prediction of SmCHS1 and SmCHS2, respectively. The amino acids belonging to the MIT domain [23] and D,D,D,QXXRW conserved motif of most processive glycosyltransferases [20] are highlighted in blue and purple, respectively. (0.55 MB JPG) [file ppat.1001070.s005.jpg]

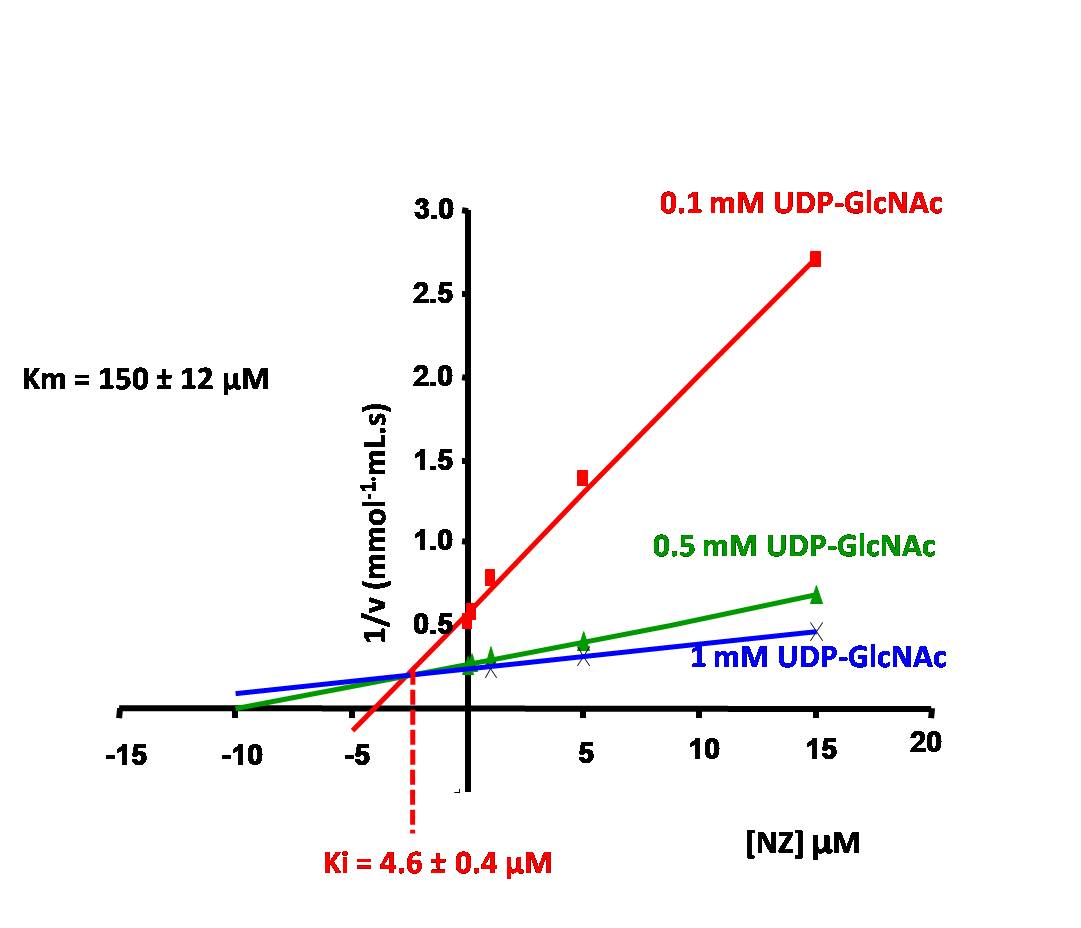

Supplement: Figure S4 — Enzyme kinetics performed on membrane fractions from S. monoica in the presence of various concentrations of UDP-GlcNAc and nikkomycin Z. The Dixon plots were used to extrapolate the values for the apparent Km and inhibition constant (Ki) of chitin synthase activity for UDP-GlcNAc and nikkomycin Z, respectively. Identical results were obtained from both the recombinant SmCHS2 (not shown) and the membrane fractions from S. monoica mycelium. (0.06 MB JPG) [file ppat.1001070.s006.jpg]

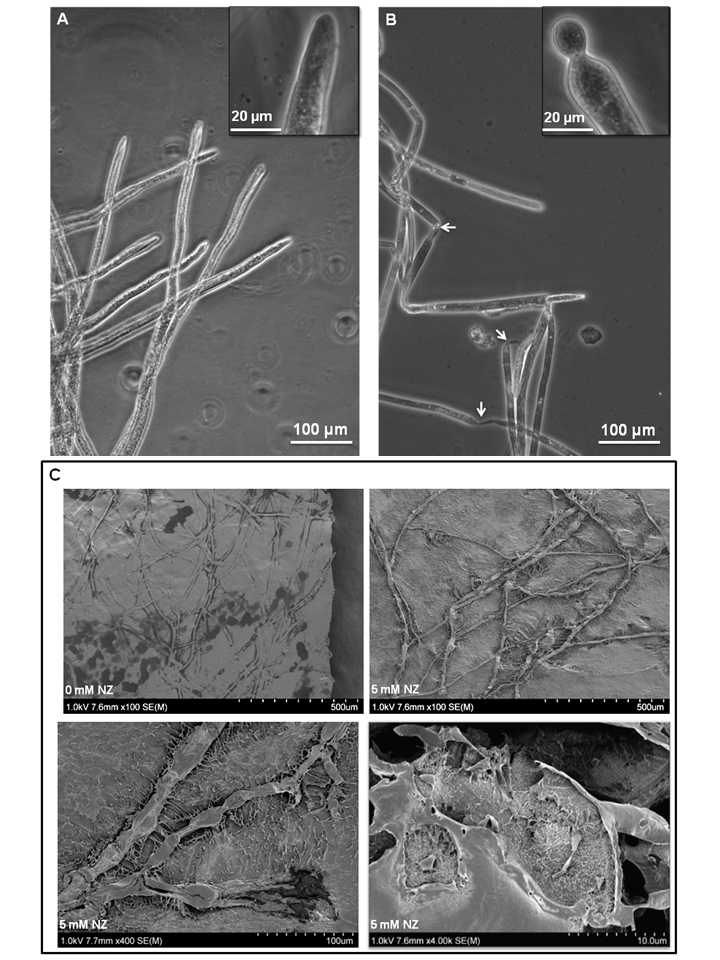

Supplement: Figure S5 — Effect of nikkomycin Z on the morphology of S. monoica mycelium. (A) Mycelium grown in the absence of inhibitor. (B) Mycelium grown for 5 days in liquid medium supplemented with 50 µM nikkomycin Z. The arrows point to morphological abnormalities. Inserts show magnifications of hyphal tips. (C) FE-SEM micrographs of hyphae grown on PDA for 5 days in the absence or presence of 5 mM nikkomycin Z. The figure shows different magnifications of the hyphae grown in the presence of nikkomycin Z. The highest magnification (bottom right micrograph) shows the morphology of a bursting hyphal tip. (0.90 MB TIF) [file ppat.1001070.s007.tif]
