# Supplementary material for: Chitin Synthases from Saprolegnia Are Involved in Tip Growth and Represent a Potential Target for Anti-Oomycete Drugs
Source: PLoS Pathog. 2010 Aug 26;6(8):e1001070. doi: 10.1371/journal.ppat.1001070 (PMC2928807; doi:10.1371/journal.ppat.1001070)
Supplement: Figure S2 — Alignment of the amino acid sequences of SmCHS1 and SmCHS2 with the Neurospora crassa and Saccharamyces cerevisiae CHS2 sequences. The CHS conserved motifs a to h are boxed. The amino acid sequence of the MIT domain (pfam 04212) is boxed and highlighted in yellow. Ser, Thr and Tyr residues that are potentially phosphorylated are shown in bold font and underlined. Potential N-glycosylation sites were identified in SmCHS1 only. They are highlighted in blue colour and bold font. Asterisks indicate residues that are identical in all sequences analysed, colons indicate conserved substitutions between the sequences and periods indicate semi-conserved substitutions. (0.09 MB PDF) [file ppat.1001070.s004.pdf]

```
S.monoicaChs1         -MPPKRPTTDGRRAYNA----------GNTTVRAPTKR--TQPRGKIGSRASNNPSAASM 47
S.monoicaChs2         -MSDQLDLAARLRALRE----------GNAAPADPEAPPPTQPAPAPQYHPQRLPPLYTQ 49
N.crassaChs2          -MDPRMHTAPPAGHP---------------------IQPGYQLEDNPYHHQQGFD-IPAG 37
S.cerevisiaeChs2      MTRNPFMVEPSNGSPNRRGASNLSKFYANANSNSRWANPSEESLEDSYDQSNVFQGLPAS 60
                                                               :       : .      :

S.monoicaChs1         QAYEFEYDYNS------------AMMPMLQ------------PPKSQPTFLNNIA-PISS 82
S.monoicaChs2         ESLEFGGTYATGSPVGAEAEGSYSQVPVWKDSKETRRSYLDDEPTPQPQSLINMANTLVQ 109
N.crassaChs2          PG----RYSPG---------DALHIQTPQP--------------IEGMGGYNAPGQHYTP 70
S.cerevisiaeChs2      PSRAALRYSPDRRHRTQFYRDSAHNSPVAPNRYAANLQESPKRAGEAVIHLSEGSNLYPR 120
                       .                        .                           .

                                                           MIT domain pfam 04212

S.monoicaChs1         KEASMKSS------------NAMQLLLQSTSFTIDDAFRAIERAIQAENEGRYREALKHF 130
S.monoicaChs2         RQASNQSFRRQHTANFRPLPNTVEELLDGTP-TYEGAFRLVQLAVQMEQDGDPGAAINLY 168
N.crassaChs2          DYAVNPEE-HHDAYYNQPYEPQVG-------HDPYAAAPTPPVAG--YQAHDDQRPMLMH 120
S.cerevisiaeChs2      DNADLPVDPYHLSPQQQPSNNLFGSGRLYSQSSKYTMSTTSTTAPSLAEADDEKEKYLTS 180
                        *                   .                    *    :

S.monoicaChs1         LDGGEMIVTAAEKEASQKVRNLLLHKGKEVLEWAEHLAEWIERYNTHSAPVRVAKPMAVE 190
S.monoicaChs2         VDAGTTLVEVGKREVDPLLQKGIQQKAFELLQRAEELGTWMNTVAEEARKAALPPQLKIA 228
N.crassaChs2          TDSQVGQS-----DPYHD---EPQPPTNNAPIKRWKTVKQVLLYRGN---------LVLD 163
S.cerevisiaeChs2      TTSYDDQSTIFSADTFNETKFELNHPTRQQYVRRANSESKRRMVSDLPPPSKKKALLKLD 240
                        .          :              :      .                    : :

S.monoicaChs1         VTYDRTMN-------SPDLDETEARTMFYTPVCCT-PQAFTETGYRLQCIQSG--RRPRL 240
S.monoicaChs2         RTNVPTVEQAWKGRTPPFHDADEFRLMRYTAVATKDPIQFSNDGYVLRVHQLH--RRIKV 286
N.crassaChs2          CPIPPKLLN-----QLPHGERDEFTHMRYSAATCD-PSEFYEENFTLRQKLFSKPRHTEL 217
S.cerevisiaeChs2      NPIPKGLLD-----TLPRRNSPEFTEMRYTACTVE-PDDFLREGYTLRFAEMN--RECQI 292
                      .    :         *  :  *   * *:.     *  * . .: *:       *. .:
                            a                                            b
S.monoicaChs1         MVVITMYNEDENELRSTLRKVCNNVLYLKQQSLPGYEGDDAWKQVLVVIVSDGRTKANKG 300
S.monoicaChs2         FITITMYNEGSEILGTLTGLAKGLGYMCKE------YGQDFWQEVAVAIVSDGRTKASKT 341
N.crassaChs2          FIVVTMYNEDEILFARTMIGVFKNIEYMCKRTESKTWGKDAWKKIVVCVVSDGRAKINPR 277
S.cerevisiaeChs2      AICITMYNEDKYSLARTIHSIMKNVAHLCKREKSHVWGPNGWKKVSVILISDGRAKVNQG 352
                       : :*****:   :  *:  : :.: :: :.      *  :  *::: *  ::****:* .

S.monoicaChs1         TLEWLSNVGLYDEDVMNITSTGVKVQCHLFEHSLQM-----TKENSIR--FPPLQVTFAL 353
S.monoicaChs2         CLEYLNGLGAFDEEIMTVTSLGVDVQMHLFESTLQL-----VENQTFENYFPPLQVIYAL 396
N.crassaChs2          TRALLAGMGVYQEGIAKQQVNGKDVTAHIYEYTTQVGMTIKNDVVQLIPKQQPVQMLFCL 337
S.cerevisiaeChs2      SLDYLAALGVYQEDMAKASVNGDPVKAHIFELTTQV--SINADLDYVSKDIVPVQLVFCL 410
                          *  :* ::* : .    *  *  *::* : *:      .   .     *:*: :.*
                                                     c
S.monoicaChs1         KEHNAGKLDSHLWYFDAFAEQVMPDYTVLIDVGTMPTKSSFYKLLTALEINAQIGGVCGE 413
S.monoicaChs2         KENNGGKLNSHLWFFNAFSEQLNPKYTVLVDVGTIPAETSVFRLIRSMERNYQIGGVAGE 456
N.crassaChs2          KEKNQKKINSHRWFFQAFGRVLDPNICVLIDAGTKPGGSSIYHLWKAFDLEPMCAGACGE 397
S.cerevisiaeChs2      KEENKKKINSHRWLFNAFCPVLQPTVVTLVDVGTRLNNTAIYRLWKVFDMDSNVAGAAGQ 470
                      **.*  *::** * *:**   : *   .*:*.**    ::.::*   :: :   .*..*:
                                           d                       e
S.monoicaChs1         IAVDKPL--PNMCNWVIAAQHFEYKISNILDKSLESCFGFISVLPGAFSAYRYKAIRG-- 469
S.monoicaChs2         IAVEA----PNYFNPVIAAQHFEYKISNIMDKSLESVFGFISVLPGAFSAYRYEAIRAVK 512
N.crassaChs2          IKAMLGTGGKNLINPLVATQNFEYKMSNILDKPLESAFGFISVLPGAFSAYRYVALQNDK 457
S.cerevisiaeChs2      IKTMKGKWGLKLFNPLVASQNFEYKISNILDKPLESVFGYISVLPGALSAYRYRALKNHE 530
                      * .       :  *  ::*:*:****:***:**.*** **:*******:***** *::
```

```
                                                                f
S.monoica Chs1          ---APLQAYFKSLTTPMAELGPFAGNMYLAEDRILCFELLARKDCNWTMHYVKDAIARTD 526
S.monoica Chs2          -GVGPLPEYFKSLTSTTKELGPFQGNMYLAEDRILCFELLARKHKQWTMHYVKDAIARTD 571
N.crassa Chs2           NGQGPLEKYFAGEKLHGGDAGIFTANMYLAEDRILCFELVTKRNCHWILQYVKSATGETD 517
S.cerevisiae Chs2       DGTGPLRSYFLGETQEGRDHDVFTANMYLAEDRILCWELVAKRDAKWVLKYVKEATGETD 590
                           .**  ** . .    : . * .***********:**::::. :* ::***.* ..**

                                  g
S.monoica Chs1          VPTNLIDLVGQRRRWLNGSFFATLFAIWNWGRVYTESNHSFTRKMALLVQYVYNVLQVIF 586
S.monoica Chs2          VPETLVDLIKQRRRWLNGSFFAGLFAIGHFGRVWSQSSHTMSRKLVFTFQFFYLALQNLL 631
N.crassa Chs2           VPADLTELILQRRRWLNGSFFAAIYAIVHFHQFFR-SDHSFLRKIAFFIEFVFQTVNMIF 576
S.cerevisiae Chs2       VPEDVSEFISQRRRWLNGAMFAAIYAQLHFYQIWK-TKHSVVRKFFLHVEFLYQFIQMLF 649
                        **  : :::  ********::** ::*  :: :.:  :.*:. **: : .::.:  :: ::

S.monoica Chs1          SWFLPANFYLALYFVIFQGFKDNRWNFIDTSKYPALLLDGLPTAFNVFYAVTVFTQVTIG 646
S.monoica Chs2          SWFLLSNLFLTFYFVLTLAFTDS---------APALLQAMLT-----LYLAIVGGLIVFA 677
N.crassa Chs2           AWFAIGNFFLVFKILTTGLGDEK---------LLGTVGQILGVVFAWAYGVTLITCFVLS 627
S.cerevisiae Chs2       SWFSIANFVLTFYYLAGSMN------------LVIKHGEALFIFFKYLIFCDLASLFIIS 697
                        :**  .*: *.:  :                       *           :   . :.

S.monoica Chs1          LGNKPKHVKGTHYLISVLFG-ILMLIASTIAIVIFV----TAHKTVEAIILAV------- 694
S.monoica Chs2          LGNKPEPRTASFYLFSCLYMGIIMMLVTGISIYGLVGKGTSAVKDPRVITGALGNCTVSE 737
N.crassa Chs2           MGNRPAGSPRLYMGMVVFWAIIFIYLMFAAIYIAVVA---IQTDVQKGLSFTDLFRNELF 684
S.cerevisiae Chs2       MGNRPQGAKHLFITSMVILSICATYSLICGFVFAFKS---LASGTES---------HKIF 745
                        :**:*      .     :                 .
                                                                            h
S.monoica Chs1          --------LILGTFFIGSAMHCEVHHIVLTFVQYTALMPSFVNILMVYSFCNLHDLSWGT 746
S.monoica Chs2          GELVGGVVTSLGLIFLSAFVHGEFS-ILLSVIQYFFMLPTFVNVLGIYAYSNLHDLSWGT 796
N.crassa Chs2           YTLIVSVVSTYGIWLIASLLMFDPWHMVTSMVQYMLLSPTYTNVLNVYAFCNTHDISWGT 744
S.cerevisiae Chs2       VDIVISLLSTYGLYFFSSLMYLDPWHMFTSSIQYFLTLPAFTCTLQIFAFCNTHDVSWGT 805
                                  *  ::.: :  :  :. : :**    *::.  * ::::.* **:****

S.monoica Chs1          KGIDTGHEHK-SDGAVGQYKD------------------IVARQKALEAKKAEDARNQDEL 788
S.monoica Chs2          KGLESGGGHGPTKTGGGNVKD------------------VVEQQKKLEAQRQAAAKEKEDV 839
N.crassa Chs2           KGDDKPDKLPSVNTKDGQGKTDLPDEGDLNASYERELQVFSRKYVKPVTAPTSAQLEEKQ 804
S.cerevisiae Chs2       KGSTQESKQLSKAIVVQGPDGKQIVETDWPQEVDKKFLEIKSRLKEPEFEESSGNEKQSK 865
                        **                  .                   .  :          .. ::.

S.monoica Chs1          KKRFDSFRSNLLLIWVMSNMAMVVICVNTIGAD-------------------SYLPFLYAF 830
S.monoica Chs2          DNSFRAFRSTLLLSWLTTNGIWLYVVTDYMSSG-------------------CYLKGLSFV 881
N.crassa Chs2           MDYYRGVRSMVVLVWMITNFALCAVVLSTAGLERIDPEEGSQEQQTTKRATIYMSVVLWS 864
S.cerevisiae Chs2       NDYYRDIRTRIVMIWMLSNLILIMSIIQVFTPQDTDNG--------------YLIFILWS 911
                         . :  .*: ::: *: :*          .                      *:  :

S.monoica Chs1          VAAFNGIRLLGCIGYLLY--YARQFLLFNTLSATGVLHKRHEARKHKKAEEDPDPIDFEM 888
S.monoica Chs2          VGFFNVIRFTGCVVFIILRIFRRFGLNCCAMGATHDTYERNLPPDWQTHYNVQNQADGRV 941
N.crassa Chs2           VAVLSGFKFVGACWFLVVRMFRGV------------------------------------ 888
S.cerevisiae Chs2       VAALAAFRVVGSMAFLFMKYLRIIVSYRNKVEGSGSWEVSKLDLPNVFHKKG-------- 963
                        *. :  ::. *.  ::.

S.monoica Chs1          GTFQNDLPDVAVPIQAPYNRMR 910
S.monoica Chs2          VVARAESINPATPRGGAYQQV- 962
N.crassa Chs2           ----------------------
S.cerevisiae Chs2       ----------------------
```
